# Hepatitis E Virus Infection in European Brown Hares, Germany, 2007–2014 

**DOI:** 10.3201/eid2506.181618

**Published:** 2019-06

**Authors:** Victor Max Corman, Laura Hilgensloh, Ulrich Voigt, Marco Marklewitz, Ursula Siebert, Christian Drosten, Jan Felix Drexler

**Affiliations:** Charité-Universitätsmedizin Berlin, Berlin, Germany (V.M. Corman, M. Marklewitz, C. Drosten, J.F. Drexler);; German Centre for Infection Research (DZIF), Berlin (V.M. Corman, C. Drosten, J.F. Drexler);; University of Bonn Medical Centre, Bonn, Germany (L. Hilgensloh);; University of Veterinary Medicine Hannover Foundation, Hannover, Germany (U. Voigt, U. Siebert)

**Keywords:** Hepatitis E, Orthohepevirus, Hares, Rabbits, Hepatitis, Viruses, Zoonoses, Germany, HEV, hepatitis E virus

## Abstract

Rabbit-associated hepatitis E viruses (HEVs) cause zoonotic infections. We investigated 2,389 hares in Germany during 2007–2014. Complete genome characterization of a hare-associated HEV strain revealed close genomic relatedness to rabbit-associated HEV strains. Although hare-specific HEV seroprevalence was low, at 2.6%, hares represent a potential source of sporadic HEV infections.

Hepatitis E virus (HEV; family *Hepeviridae,* genus *Orthohepevirus*) is a major cause of acute hepatitis in humans worldwide. Large outbreaks have included waterborne HEV in the tropics and zoonotic infections in temperate climates (*1*). In 2017, researchers identified human infection with HEV strains associated with rabbits (raHEV) in France (*2*,*3*). 

In Germany, rabbits (family *Leporidae,* genus *Oryctolagus*) are common in the wild but are also popular pets, potentially explaining human exposure to raHEV. Hares (family *Leporidae,* genus *Lepus*), although genetically related, are physically larger and are primarily hunted as food in large parts of Europe (>5 million hares annually) (*4*). HEV associated with hares (haHEV) had been previously unknown (*5*). For this study, we tested for HEV in 2,389 European brown hares (*L. europaeus*) hunted in Germany during 2007–2014, across an area of ≈30,000 km^2^ (Appendix Figure 1). One animal (0.04% of all samples; 95% CI 0.03%–0.05%) tested positive in a broadly reactive reverse transcription PCR assay; 25 (2.6%; 95% CI 1.6%–3.5%) animals were identified as seropositive. 

RNA concentration was 5.4 × 10^4^ IU/mL in the serum of the animal that tested positive, comparable to viral loads observed in rabbits and in human blood donors (*3*,*6*). After we determined its complete viral genome (GenBank accession no. MK050463), the haHEV isolate showed the typical genome organization of *Orthohepevirus* A, including 3 predicted open reading frames (ORF). We found a 93-nt insertion in the X-domain of ORF1 that is thought to be typical of raHEV strains and absent in other HEV strains (*2*,*3*). This finding may reflect a common origin of raHEV and haHEV strains or a host-associated virus adaptation. We found no evidence for recombination of haHEV with other *Orthohepevirus* species or subtypes. 

When averaged over the complete genome, the haHEV isolate we obtained shared 86% nucleotide identity with the most closely related raHEV strain (GenBank accession no. KY436898), from a rabbit sampled in Germany in 2016 (*5*). Average translated amino acid identity of the haHEV with raHEV strains was high at 88.5%–97.5% for ORF1, 90.2%–98.5% for ORF2, and 88.6%–95.1% for ORF3. In phylogenetic reconstructions, the haHEV clustered with raHEV strains, further suggesting a common origin for haHEV and raHEV, and suggesting an origin of haHEV in ancestors carried by rabbits and distinct from the greater HEV diversity existing in swine (*7*) (Figure; Appendix Figure 2). This interpretation is consistent with sporadic sharing of habitats between wild rabbits and hares in the study area. 

**Figure:**
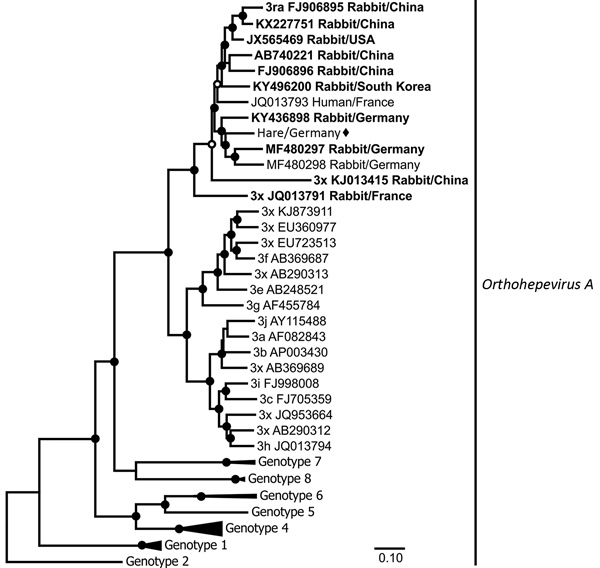
Maximum-likelihood phylogeny of the concatenated open reading frame 1 and 2 nucleotide sequences of hare hepatitis E virus (HEV) strain from Germany (black diamond), related strains from rabbits (bold) and humans, and reference *Orthohepevirus A* strains, as defined by Smith et al. (*7*). Taxon names of all reference sequences include genotype, subtype (x if not available), and GenBank accession number. Black circles at nodes indicate bootstrap supports of >90% and white circles >75% (1,000 replicates). The clades comprising sequences other than HEV genotype 3 were collapsed for graphical reasons. Scale bar indicates nucleotide substitutions per site.

To determine seroprevalence, we expressed the capsid protein of haHEV and used it for indirect immunofluorescence assays (Appendix). In total, serum samples from 944 hares, covering all sampling regions and years, were available in sufficient quality and quantity for serological testing (Appendix Table 2). HEV seroprevalence for hares was 2.6% (95% CI 1.6%–3.5%), similar to values in a previous serologic study of 669 European brown hares that used a capsid fragment from a human HEV genotype 1 strain as antigen and yielded a seroprevalence of 1.6%–4.3% (*5*). The sampling sites from that study were up to 400 km from those in our study, suggesting geographically widespread HEV infection of hares. That study also found wild rabbits to be seropositive at rates >35% (*5*). The lower HEV seroprevalence in hares than in rabbits might be explained by the fact that hares live solitarily and have low-density populations, ≈12–20 animals/100 ha in the sampling region; wild rabbits live in groups of ≈15 that are also proximal to other groups. 

We found no statistically significant differences in seroprevalence for hares <1 versus >1 year of age (χ^2^ 0.08; p = 0.78), which is unlike the age-dependent increase of seropositivity observed in pigs and humans (*8*,*9*). Explanations may include a lack of statistical power in this study or differential pathogenesis of HEV in hares. Although the data on HEV pathogenesis in rabbits are in part controversial, occurrence of HEV in apparently healthy laboratory rabbits suggests that rabbits frequently survive HEV infection (*10*). Whether the apparently low seroprevalence in hares compared with rabbits is thus due to infrequent infection, differential antibody responses, or other host- or virus-associated factors remains to determined.

We detected no statistically significant differences in seroprevalence rates, either between sexes (χ^2^ 0.01; p = 0.92) or across the 8 sampling years (Yates χ^2^ 0.6; p = 0.96) and the 5 individual sampling regions (Yates χ^2^ 1.945; p = 0.96). These findings suggested constant low levels of HEV transmission in hares irrespective of sex and geographic region.

The infection of hares with HEV strains that are closely related to raHEV strains suggests that hares may act as sporadic sources of zoonotic HEV infections. Although the low RNA detection rate and seroprevalence speak against a prominent role of hares in the epidemiology of zoonotic HEV, hunters and persons handling hare-derived products could represent risk groups. Awareness about hare-derived HEV infections may be particularly relevant for immunocompromised persons, in whom chronic HEV infections are most common.

Appendix Details of methodology for sampling; viral nucleic acid detection and complete genome sequencing; and antibody detection
