# Supplementary material for: Hepatitis E Virus Infection in European Brown Hares, Germany, 2007–2014
Source: Emerg Infect Dis. 2019 Jun;25(6):1233–5. doi: 10.3201/eid2506.181618 (PMC6537731; doi:10.3201/eid2506.181618)
Supplement: Appendix — Details of methodology for sampling; viral nucleic acid detection and complete genome sequencing; and antibody detection [file 18-1618-Techapp-s1.pdf]

Article DOI: https://doi.org/10.3201/eid2506.181618

Hepatitis E Virus Infection in European Brown Hares, Germany 2007–2014

Appendix

Sampling

A total of 2,389 serum specimens obtained from European brown hares (*Lepus europaeus*) were available for this study (Appendix Table 1). Samples were collected from dead hares during hunting days in 25 counties, summarized into 5 regions of Lower Saxony between 2007 and 2014 (Appendix Figure 1). Hare blood was sampled by heart punctuation shortly after death. Serum samples were obtained after centrifugation of coagulated blood sample at 3,000 × g for 15 minutes. All samples were stored at –20°C until further processing. Data on age of 880 hares was determined by the mean dry weight of the eye lenses (*1*). Here, we used 280 mg as the borderline value of mean eye lenses weight between juvenile (<1 year) and adult hares (>1 year) as described elsewhere (*1*). Data about hare and rabbit density and ecology were obtained from the annual hunting reports published from the Ministry of Food, Agriculture, and Consumer Protection, Lower Saxony (*1*) and from own observations.

Viral nucleic acid detection and complete genome sequencing

Viral RNA was extracted with the QiaCube HT (QIAGEN, Hilden, Germany) using 20µl of serum from all 2,389 individual hares. Screening for hepatitis E virus (HEV) RNA was done using a highly sensitive and broadly reactive nested reverse transcription-PCR (RT-PCR) assay (oligonucleotides are given in Appendix Table 3) amplifying a 283 nt fragment of the *RNA-dependent RNA-polymerase* gene as previously described (*2*). The amplified RT-PCR screening fragment was Sanger sequenced (Seqlab Göttingen, Germany).

The HEV RNA concentration in the positive sample was determined by using a real-time RT-PCR assay (oligonucleotides are given in Appendix Table 3) designed for the detection and quantification of *Orthohepevirus A* (*3*). The World Health Organization International Standard for HEV (*4*) was used for calibration.

To obtain the full sequence of the hare HEV genome, heminested reverse transcription-PCR (RT-PCR) assays were developed (Appendix Table 3). These assays were designed to amplify ≈800 overlapping nucleotides of known *Orthohepevirus A* genotype 3 genomes. Genomic fragments that could not be amplified by these assays were connected by bridging RT-PCR using hare HEV-specific primers (Appendix Table 3). All PCR products were Sanger sequenced (Microsynth Seqlab, Göttingen, Germany).

For the phylogenetic analyses, the complete coding sequences of the concatenated ORF 1 and ORF 2 of hare-associated HEV were aligned with other lagomorph HEV strains available in GenBank as of 1st Sep 2018, and reference *Orthohepevirus A* strains, as previously defined (*5*). Maximum likelihood phylogenies were calculated using a GTR nucleotide substitution model, neighbor-joining phylogenies with a Jukes-Cantor nucleotide substitution model and 1000 bootstrap replicates in Geneious 11 (https://www.geneious.com/). Recombination analyses were made using RDP4 (*6*).

Antibody detection

Recombinant capsid Immunofluorescence Assay (IFA)

For expression of the viral capsid protein, the hare HEV capsid gene obtained from complete genome sequencing was codon-optimized for expression in primate cells (http://eu.idtdna.com/CodonOpt) and synthesized (Integrated DNA Technologies, Leuven, Belgium) including a C-terminal FLAG-tag. The synthesized gene was cloned into a pCG1 eukaryotic expression vector (kindly provided by Georg Herrler, University of Veterinary Medicine Hannover, Foundation, Hanover) and its sequence confirmed by re-sequencing. Vero B4 cells were transfected in suspension using 2.5 μg of plasmid DNA and the FuGENE HD protocol (Roche, Basel, Switzerland). Transfected cells were seeded into a 6-well plate. After 24 hours, cells were washed, trypsinized and transferred to multitest glass slides (Dunn Labortechnik, Asbach, Germany). After 6 hours, cells were fixated with ice cold acetone/methanol and stored dry at 4°C. As the C-terminal flag tag will be only expressed when the upstream viral gene is intact, we used a mouse anti-FLAG antibody (Sigma-Aldrich Chemie GmbH), diluted 1:100 and a Cy3-labeled goat anti-mouse antibody, diluted 1:200 (Jackson ImmunoResearch Laboratories, Ely, UK) to confirm expression of viral proteins.

For validation of our recombinant capsid IFA, we used five human sera shown to be positive for anti-Hepatitis E IgG by a human-specific Anti-HEV IgG ELISA (Axiom Diagnostics Worms, Germany) and the HEV recomLine Immunoblot (MIKROGEN, Neuried, Germany) as well as a serial dilution (50 IU/ml down to 0.5 IU/ml) of the WHO reference reagent for antibodies to hepatitis E virus (NIBSC code: 95/584) (*7*). Human serum samples and the reference reagent were applied at a dilution of 1:40 and detection involved a goat anti-human IgG coupled with Alexa 488 (Dianova, Hamburg, Germany) diluted 1:200. All five sera reactive in ELISA were also positive in the hare HEV capsid-based IFA. Five human sera testing negative in the IgG ELISA showed no signal in the hare HEV capsid-based IFA (Appendix Figure 3). The WHO reference reagent showed positive signal down to a concentration of 1 IU/ml in the IFA (Appendix Figure 4). To further validate the recombinant capsid IFA for hare-derived samples, we first tested the HEV RNA positive hare sample by using the recombinant IFA slides and by using different dilutions (1:40 to 1:4,000) of the hare serum, the secondary (AffiniPure Goat Anti-Rabbit IgG (H+L); Jackson ImmunoResearch Laboratories) and tertiary antibody Cy3 labeled-AffiniPure Donkey Anti-Goat IgG (H+L; Jackson ImmunoResearch Laboratories) in the range of 1:100 to 1:2,000. The secondary and tertiary antibodies were found to show best signals without background noise at a dilution of 1:1,500 and 1:200, respectively. The absence of any signal by using the tertiary antibody without preincubation of the secondary (Goat Anti-Rabbit IgG) excludes unspecific signal of the tertiary antibody (Donkey Anti-Goat IgG) with the cells. For hare HEV antibody screening, hare serum samples were applied at a dilution of 1:40 for 1 h at 37°C. As an example, the IFA of the HEV RNA positive and IFA reactive hare serum sample is shown in Appendix Figure 5. Untransfected cells served as internal negative controls.

References

1. Gräber R, Strauß, E. und S. Johanshon. Wild und Jagd–Landesjagdbericht. Niedersächsisches Ministerium für Ernährung, Landwirtschaft und Verbraucherschutz. 2013;2012/13 [cited February 1, 2019]. https://www.ml.niedersachsen.de/download/82676/Landesjagdbericht_2012_2013.pdf

2. Drexler JF, Seelen A, Corman VM, Fumie Tateno A, Cottontail V, Melim Zerbinati R, et al. Bats worldwide carry hepatitis E virus-related viruses that form a putative novel genus within the family Hepeviridae. J Virol. 2012;86:9134–47. http://dx.doi.org/10.1128/JVI.00800-12

3. Girón-Callejas A, Clark G, Irving WL, McClure CP. In silico and in vitro interrogation of a widely used HEV RT-qPCR assay for detection of the species Orthohepevirus A. J Virol Methods. 2015;214:25–8. http://dx.doi.org/10.1016/j.jviromet.2014.11.025

4. Baylis SA, Blümel J, Mizusawa S, Matsubayashi K, Sakata H, Okada Y, et al.; HEV Collaborative Study Group. World Health Organization International Standard to harmonize assays for detection of hepatitis E virus RNA. Emerg Infect Dis. 2013;19:729–35. http://dx.doi.org/10.3201/eid1905.121845

5. Smith DB, Simmonds P, Izopet J, Oliveira-Filho EF, Ulrich RG, Johne R, et al. Proposed reference sequences for hepatitis E virus subtypes. J Gen Virol. 2016;97:537–42. http://dx.doi.org/10.1099/jgv.0.000393

6. Martin DP, Murrell B, Golden M, Khoosal A, Muhire B. RDP4: Detection and analysis of recombination patterns in virus genomes. Virus Evol. 2015;1:vev003. PubMed https://doi.org/10.1093/ve/vev003.

7. Ferguson M, Walker D, Mast E, Fields H. Report of a collaborative study to assess the suitability of a reference reagent for antibodies to hepatitis E virus. Biologicals. 2002;30:43–8. http://dx.doi.org/10.1006/biol.2001.0315

Appendix Table 1. Number and origin of hare serum samples used for HEV RT-PCR screening*

**Sample origin**		**Year of sampling**								
**Region**	**County**	**2007**	**2008**	**2009**	**2010**	**2011**	**2012**	**2013**	**2014**	**Total**
**CE**		**56**	**70**	**53**	**27**			**7**		**213**
	Celle		21							21
	Diepholz			39	27					66
	Hannover, Region	56		14				7		77
	Nienburg		35							35
	Wolfsburg		14							14
**CW**		**73**	**54**	**62**						**189**
	Cloppenburg		13							13
	Emsland	38	13							51
	Oldenburg			54						54
	Osnabrück	35								35
	Vechta		28	8						36
**NE**			**113**	**14**						**127**
	Harburg		16	14						30
	Lüneburg		22							22
	Rotenburg		30							30
	Stade		14							14
	Verden		31							31
**NW**		**3**	**111**	**99**	**92**					**305**
	Ammerland		51							51
	Aurich		19							19
	Osterholz		10							10
	Wesermarsch			4	51					55
	Wittmund	3	31	95	41					170
**SE**		**257**	**357**	**224**	**483**	**54**	**158**	**10**	**12**	**1,555**
	Goslar	5								5
	Göttingen	33								33
	Hildesheim		39		33					72
	Northeim	40								40
	Peine	179	318	224	450	54	158	10	12	1,405
	**Total**	**389**	**705**	**452**	**602**	**54**	**158**	**17**	**12**	**2,389**

*CE, Central-East; CW, Central-West; NE, North-East; NW, North-West, SE, South-East

Appendix Table 2. Number and origin of hare serum samples used for antibody testing (number of IFA positive samples)*

**Sample origin**	**Year of sampling**								
**Region**	**2007**	**2008**	**2009**	**2010**	**2011**	**2012**	**2013**	**2014**	**Total (pos.)**
CE	29	66 (5)	44 (1)	21			7		167 (6)
CW	60	54 (2)	38 (2)						152 (4)
NE		113 (2)	14						127 (2)
NW	3	89 (1)	34	60 (3)					186 (4)
SE	90 (3)	60 (1)	30	60 (1)	30 (2)	30 (1)		12 (1)	312 (9)
**Total (pos.)**	**182 (3)**	**382 (11)**	**160 (3)**	**141 (4)**	**30 (2)**	**30 (1)**	**7**	**12 (1)**	**944 (25)**

CE, Central-East; CW, Central-West; NE, North-East; NW, North-West, SE, South-East

Appendix Table 3. Oligonucleotides used for RT-PCR screening, complete genome sequencing, and virus quantification

Virus targeted	Oligonucleotide	Sequence (5' – 3')*	Use	Reference
Orthohpeviruses A–D	HEV-F4228	ACYTTYTGTGCYYTITTTGGTCCITGGTT	Heminested screening RT-PCR	(2)
	HEV-R4598	GCCATGTTCCAGAYGGTGTTCCA		
	HEV-R4565	CCGGGTTCRCCIGAGTGTTTCTTCCA		
Orthohepevirus A	OrthoHEV_A-rtF	GGTGGTTTCTGGGGTGAC	Quantitative real-time RT-PCR	(3)
	OrthoHEV_A-rtP	FAM-TGATTCTCAGCCCTTCGC-MGB		
	OrthoHEV_A-rtR	AGGGGTTGGTTGGRTGRA		
Orthohepevirus A genotype 3	HEV-F4228	ACYTTYTGTGCYYTITTTGGTCCITGGTT	Heminested RT-PCRs for full-genome sequencing	This study
	HEVgt3–4611F	GGAAGAAGCAYTCYGGTGAGC		
	HEVgt3–5305F	GTGGTTTCTGGGGTGACAGG		
	HEVgt3–5325Fn	GTTGATTCTCAGCCCTTCGC		
	HEVgt3–5349Rn	GGTTGGTTGGATGAATATAGGG		
	HEVgt3–5448R	GCTGGGACTGGTCRCGCCA		
	HEVgt3–5958F	GGNTGGCGCTCNGTNGAGAC		
	HEVgt3–6000R	AGCATTACCAGRCCRGARGTAGC		
	HEVgt3–6341F	GACAGAATTRATTTCGTCGGC		
	HEVgt3–6341Rn	GCCGACGAAATYAATTCTGTC		
	HEVgt3–6378Fn	TACTCCCGCCCRGTYGTCTC		
	HEVgt3–6393R	GCTCGCCATTGGCYGAGAC		
	HEVgt3–7145R	TCCCGRGTTTTRCCYACCTTCA		
Hare/Rabbit HEV	Hare3Screen-F	CTAATTCGGTCGACCTGGATCC	Heminested RT-PCRs for full-genome sequencing	This study
	HaHEV3Screen-Fn	GGATCCTACAGGCTCCAAAGG		
	HaHEV-R4565	CCGGGTTCRCCIGAGTGTTTCTTCCA		
	HaHEV-R4598	GCCATGTTCCAGAYGGTGTTCCA		
	HaHEV-4979F	CAGTTACGCTTGGCTGTTTGC		
	HaHEV-F 5212	GCATCGCCCATGGGTTCAC		
	HEVgt3P3-R	TGBAGCATRCCRATAAGGTTATG		
	HEVgt33-Rnest	TARACHCGVGAMACAACATCMAC		
	HaHEV-5255F	GCTGTTCGTCGTGTGTTTGC		
	HaHEV-5142Fs	CTGTCAAGCCTGTGTTAGACC		
	HaHEV-5050F	GTCCCGTGTTTATGGTGTGAGC		
	HaHEV-6162F	CTTGAGATTGAGTTCCGCAACC		
	HaHEV-6186F	ACTCCAGGGAACACCAACACAC		
	HaHEV-6321R	TCTCACCAACCCCGTTCATCC		
	HaHEV-6347R	TAATGTCAGGGCTATGCCACG		
	HaHEV-7231R	GGCACAACAAGAATTAATTAAAACTCC		
	HaHEV-7246R	GCAATATAGAAGGGGGCACAAC		
	HaHEV-3R-F	CTGTCTCTATCTCTGCAGTCG		
	HaHEV-3R-Fn	CTGTTCTAGCTGTCCTTGAGG		
	HaHEV-3R-Fs	CTAGCTGTCCTTGAGGATACTACTG		

*Equally mixed base ratios in the sequences are represented as standard code letters: R is G/A, Y is C/T, S is G/C,Wis A/T, M is A/C, K is G/T, H is A/C/T, B is C/G/T, N is A/T/C/G, and I is inosine. FAM, 6-carboxyfluorescein; MGB, minor groove binder.

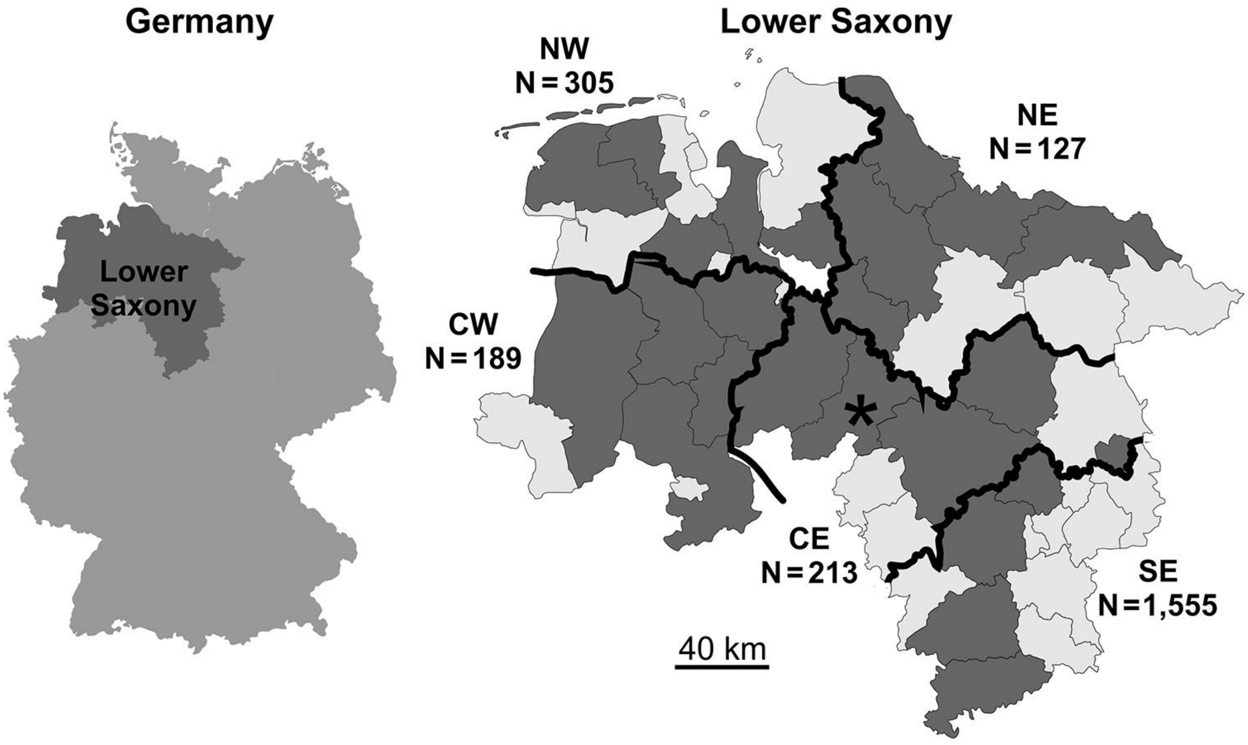

Appendix Figure 1. Number of hare samples available for molecular testing. The county where the HEV RNA positive sample was detected is marked with an asterisk. Map was created by using Quantum GIS (http://qgis.osgeo.org). CE, Central-East; CW, Central-West; NE, North-East; NW, North-West, SE, South-East.

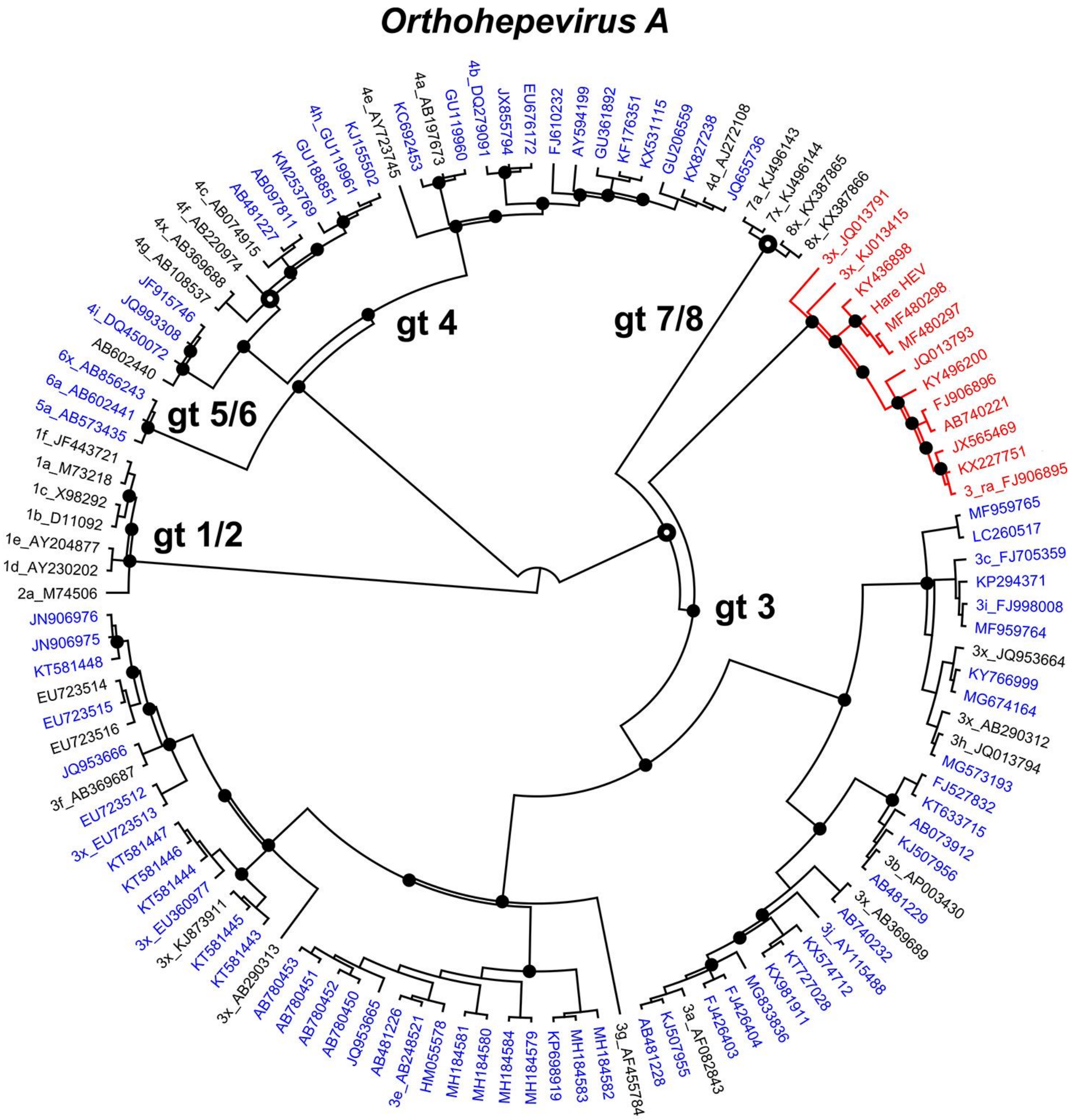

Appendix Figure 2. Neighbor-joining phylogeny of the concatenated ORF 1 and 2 nt sequences of the lagomorph-associated HEV strain, reference sequences for *Orthohepevirus A* strains/subtypes, as defined by Smith et al. (*5*), and all complete genome sequences from pigs and wild boars available in GenBank as of 1st January 2019. Taxon names of all reference sequences include genotype, subtype (“x” if not available), and GenBank accession number. Black circles at deeper nodes indicate bootstrap supports of >90% and white circles >75% (1000 replicates). All sequences obtained from swine are given blue. gt, genotype

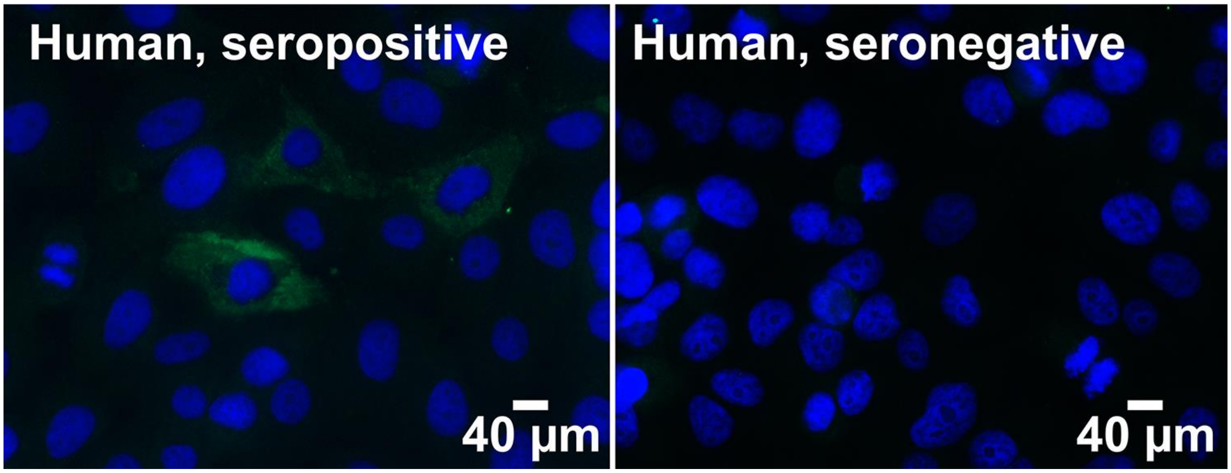

Appendix Figure 3. Example of an anti-hepatitis E IgG positive and negative human serum sample (serum dilution, 1:40) in the recombinant hare HEV capsid-based indirect immunofluorescence assay. HEV antibodies are tagged with a green fluorophore. Nuclei are stained with DAPI (blue).

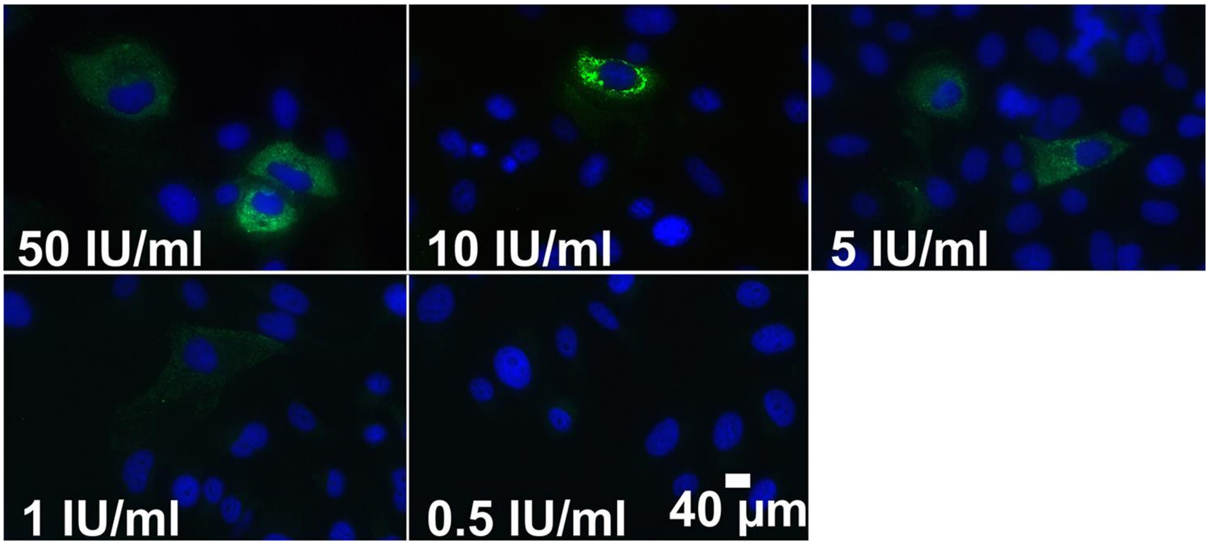

Appendix Figure 4. Serial dilution (50 IU/ml down to 0.5 IU/ml) of the WHO reference reagent for antibodies to hepatitis E virus (NIBSC code: 95/584) tested in the recombinant hare HEV capsid-based indirect immunofluorescence assay. HEV antibodies are tagged with a green fluorophore. Nuclei are stained with DAPI (blue).

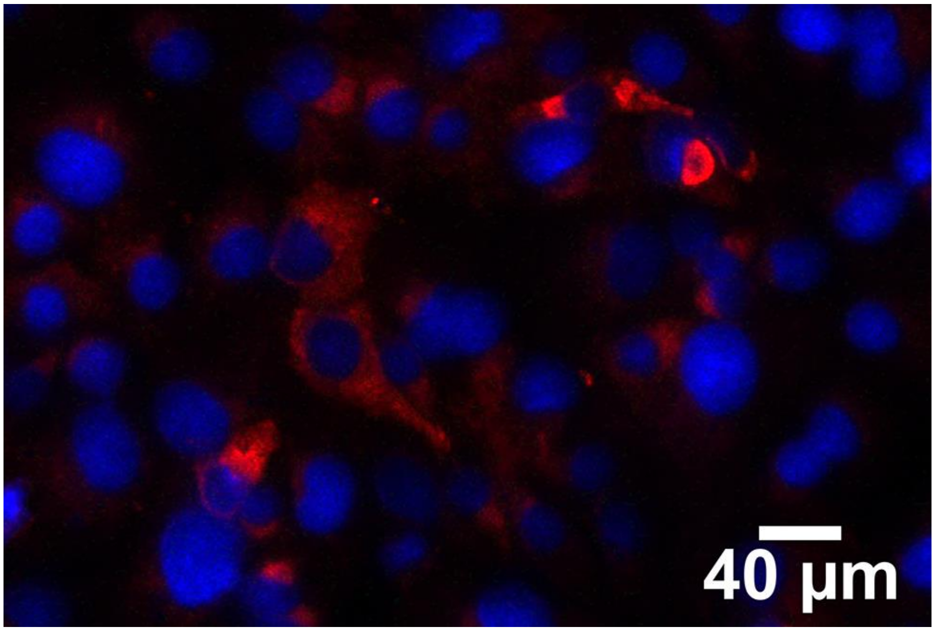

Appendix Figure 5. Example of a HEV-reactive hare serum sample (serum dilution, 1:40) in the recombinant hare HEV capsid-based indirect immunofluorescence assay. HEV antibodies are tagged with a red fluorophore. Nuclei are stained with DAPI (blue).
